# Metabonomic analysis of abnormal sphingolipid metabolism in rheumatoid arthritis synovial fibroblasts in hypoxia microenvironment and intervention of geniposide

**DOI:** 10.3389/fphar.2022.969408

**Published:** 2022-07-22

**Authors:** Jiang-Tao Ke, Heng Zhang, Yan-Hong Bu, Pei-Rong Gan, Fang-Yuan Chen, Xin-Tong Dong, Yan Wang, Hong Wu

**Affiliations:** ^1^ Key Laboratory of Xin’an Medicine, Ministry of Education, Hefei, China; ^2^ College of Pharmacy, Anhui University of Chinese Medicine, Hefei, China; ^3^ Anhui Province Key Laboratory of Research & Development of Chinese Medicine, Hefei, China; ^4^ Anhui Province Key Laboratory of Chinese Medicinal Formula, Hefei, China

**Keywords:** rheumatoid arthritis, sphingolipid, metabolomics, geniposide, UPLC-Q-TOF/MS

## Abstract

Rheumatoid arthritis (RA) is a chronic inflammatory disease characterized by a joint hypoxia microenvironment. Our previous untargeted metabolomics study found that sphingolipid (SPL) metabolism was abnormal in the joint synovial fluid samples from adjuvant arthritis (AA) rats. Geniposide (GE), an iridoid glycoside component of the dried fruit of *Gardenia jasminoides* Ellis, is commonly used for RA treatment in many Asian countries. At present, the mechanism of GE in the treatment of RA, especially in the joint hypoxia microenvironment, is not entirely clear from the perspective of SPL metabolism. The purpose of this research was to explore the potential mechanism of abnormal SPL metabolism in RA joint hypoxia microenvironment and the intervention effect of GE, through the untargeted metabolic analysis based on the ultra-performance liquid chromatography-quadrupole time-of-flight mass spectrometry (UPLC-Q-TOF/MS). Arthritis index, foot swelling and histopathology were used to assess whether the AA rat model was successfully established. The SPLs extracts collected from AA rats’ synovial tissue, serum and rheumatoid arthritis synovial fibroblasts (RASFs, MH7A cells, hypoxia/normoxia culture) were analyzed by metabolomics and lipdomics approach based on UPLC-Q-TOF/MS, to identify potential biomarkers associated with disorders of GE regulated RA sphingolipid metabolism. As a result, 11 sphingolipid metabolites related to RA were screened and identified. Except for galactosylceramide (d18:1/20:0), GE could recover the change levels of the above 10 sphingolipid biomarkers in varying degrees. Western blotting results showed that the changes in ceramide (Cer) level regulated by GE were related to the down-regulation of acid-sphingomyelinase (A-SMase) expression in synovial tissue of AA rats. To sum up, this research examined the mechanism of GE in the treatment of RA from the perspective of SPL metabolism and provided a new strategy for the screening of biomarkers for clinical diagnosis of RA.

## Introduction

Rheumatoid arthritis (RA) is a common autoimmune disease whose main pathological features are abnormal hyperplasia of synovial tissue, persistent synovial inflammation, angiogenesis and pannus formation (Scott et al., 2010). The inflammatory response is the most common stress response when tissue is damaged, which plays an important role in the occurrence and development of RA (Wang L. et al., 2021). A large number of inflammatory factors such as hypoxia-inducible factor-1α (HIF-1α), tumor necrosis factor-α (TNF-α) and interleukin-1β (IL-1β) filled with synovitis microenvironment, they directly broke the balance of normal synovial metabolism (Qu et al., 2021; Tsuchiya et al., 2021). Rheumatoid arthritis synovial fibroblasts (RASFs) are the main effector cells of RA. Under the pathological state, they show the phenomenon of excessive proliferation and insufficient apoptosis, which makes the synovial tissue proliferate and hypertrophy, increases the oxygen consumption and produces an “anoxic microenvironment”, resulting in the formation of a large number of synovial microvessels with abnormal structure and function, and aggravates synovial inflammation (Chen et al., 2021). In recent years, an increasing number of studies have shown that hypoxia-mediated metabolic disorders play an important role in the pathogenesis of RA. The finding of drugs that regulate metabolic disorders may help improve the disease process in RA patients.

Geniposide (GE), a kind of iridoid compound, is one of the main active components in *Gardenia jasminoides* Ellis, with anti-inflammatory, anti-allergic and immunosuppressive and other pharmacological effects (Deng et al., 2021). In Our previous studies, untargeted metabolomic analysis based on hydrophilic interaction liquid chromatography coupled to high-resolution mass spectrometry (HILIC-HRMS) was performed using the joint synovial fluid samples from adjuvant arthritis (AA) rats. It was found that 20 metabolites differed significantly between AA rats and normal rats, which were mainly involved in glycerophospholipid metabolism, sphingolipid metabolism and purine metabolism. GE could reverse the levels of 13 metabolites in AA rats, including palmitoyl ethanolamine (PEA), ceramide (Cer) and phenylacetic acid, and showed the ability to regulate unbalanced sphingolipid metabolism (Zhan et al., 2020). In addition, GE alleviated inflammation in AA rats by modulating the sphingolipid metabolic pathway and reducing the levels of sphingomyelin (SM) in jugular vein micro-dialysates and Cer and D-sphingomyelin (DSA) in knee micro-dialysates (Wang R. H. et al., 2020; Wang M. et al., 2021). In conclusion, the above studies showed that GE can regulate the biological level of Sphingolipids (SPLs) in AA rats.

Hypoxia is one of the key features of the intra-articular microenvironment in RA (Deng et al., 2022). In addition, SPLs are of interest as important cellular mediators of inflammatory hypoxia, and hypoxia is capable of disrupting sphingolipid metabolism in different organisms (e.g., increased SIP levels and decreased Cer levels in vascular smooth muscle cells under hypoxia conditions) to accelerate disease progression (Yun and Kester, 2002; Ottolenghi et al., 2020). SPLs are the most abundant class of bioactive lipids in living organisms, which are essential regulators of physiological and pathological processes including cell cycle, angiogenesis and inflammatory responses (Hannun and Obeid, 2018). There are two main production modes: sphingomyelin metabolism and *de novo* synthesis of serine and palmitoyl acyl-CoA (Mortimer and Scheller, 2020). Sphingolipid metabolites, especially Cer, sphingosine and sphingosine 1-phosphate (S1P), are important signal molecules in cell signal transduction, cell proliferation and apoptosis. These cellular processes are highly crucial in immune-inflammatory diseases (Maceyka and Spiegel, 2014; Skácel et al., 2021). Recently, people have a new understanding of the molecular mechanism of sphingolipid metabolites and their regulation of chronic inflammation, which will be more conducive to the development of new treatments for inflammatory diseases.

Metabolomics, the research of small-molecule metabolites (relative molecular mass less than 1000) in complex biological systems, is a plausible approach to elucidate the full range of metabolic changes induced by pathophysiological stimuli to biological systems (Jang et al., 2018). In particular, with the advantages of high resolution, high sensitivity, high reproducibility and less sample requirements, UPLC-Q-TOF/MS has become the tool of choice for studying disease-related changes in metabolic pathways and for the discovery of endogenous metabolites (Rinschen et al., 2019; Wang et al., 2019; Yin et al., 2022). The investigation of functional endogenous metabolites in biological systems using HRMS combined with multivariate statistical analysis to identify potential biomarkers associated with the disease can provide valuable information about the pathological mechanisms and pharmacological interventions in complex diseases (Zhao et al., 2018). Lipidomics, as a separate discipline from metabolomics, specializes in the study of lipid changes in living organisms under physiological pathology and provides comprehensive lipid information in biological samples (Wang R. et al., 2020). Currently, metabolomics and lipidomics have been widely used in the study of the mechanism of active molecules in traditional Chinese medicine (TCM) (Wu et al., 2020). Numerous metabolomics studies have shown that the abnormal metabolism of RA is related to sphingolipid metabolism. Therefore, the integration of metabolomics and lipidomics can reveal the sphingolipid biomarkers of RA treatment in a more comprehensive and integrated manner.

In this study, we established a rat’s model of adjuvant arthritis (AA) and a hypoxia model of MH7A cells using a hypoxia incubator to simulate the hypoxia microenvironment in the joint cavity of RA patients. Subsequently, a UPLC-Q-TOF/MS based metabolomics and lipidomics platform was employed to investigate the potential sphingolipid markers in AA rat synovial tissue, serum, and hypoxia cultured MH7A cells to reveal the mechanism of GE treatment for RA from the perspective of abnormal metabolism of SPLs.

## Materials and methods

### Materials and reagents

**FIGURE 1:**
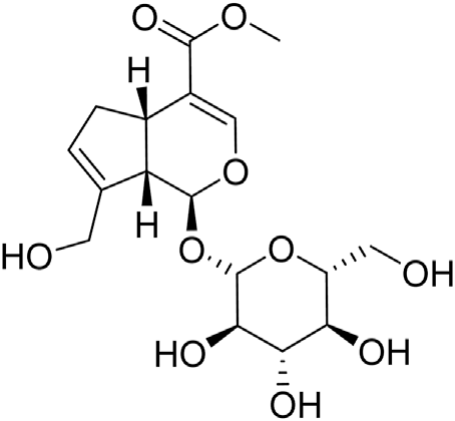
Chemical structure of geniposide.

GE (C_17_H_24_O_10_, molecular weight: 388, Purity >98%, batch number: S25627, structure shown in Figure 1) and Urethane (batch number: 20200710) were purchased from the National Institute for the Control of Pharmaceutical and Biological Products (Beijing, China). Methotrexate (MTX) (batch number: B25455) was provided by Yuanye Biotechnology Co., Ltd. (Shanghai, China). Formic acid (batch number: 100264) was obtained from Merck Chemical Technology Co., Ltd. (Shanghai, China). Freund’s Complete Adjuvant (FCA) (batch number: F5881) was obtained from Sigma-Aldrich (St. Louis, MO, United States ). LC-MS grade acetonitrile (batch number: A104443), methyl tert-butyl ether (MTBE, batch number: B108120), Methanol (batch number: M116126), and Isopropanol (batch number: I112020) were provided by Aladdin Bio-Chem Technology Co., Ltd. (Shanghai, China). Ultrapure water was produced by a Milli-Q Gradient A10 Ultrapure Water System (MILLIPORE Co., Ltd., United States ). Dulbecco’s modified Eagle’s medium (DMEM) for MH7A cells culture was purchased from Hy Clone (Logan, UT, United States ). Fetal bovine serum (FBS) was provided by Biological Industries Co., Ltd. (Kibbutz BH, Israel).

### Establishment and evaluation of adjuvant arthritis rat model

Male Sprague-Dawley rats (180–220 g, Grade Ⅱ) were provided by the Experimental Animal Center of Anhui University of Traditional Chinese Medicine (Hefei, China). The rats were housed under the conditions of temperature at 25 ± 1°C and 45–55% relative humidity for 12 h light/12 h darkness cycles. All rats were kept under this condition for at least one week before the experiment. The experimental protocol was approved by the Animal Ethics Committee of Anhui University of Traditional Chinese Medicine (No. AHTCM-rats-2021049) and all experimental methods were carried out by following the animal experiment operation guidelines. The rat’s left hind toe received a subcutaneous injection of 100 µL of FCA on day 0, and the control group rats were injected with 100 µL of physiological saline at the same time (Deng et al., 2018). On the 16th day, the rats were randomly divided into four groups (n = 24): normal control group, model group, GE (60 mg/kg/day, 14 days, intragastrically (ig)) group and MTX (positive medicine) group (0.5 mg/kg, three times a week, 14 days, ig), on day 7, day 14, day 17, day 20, day 23, day 26, day 29, the arthritis index and secondary paw swelling of the four groups of rats were evaluated. The arthritis index and secondary paw swelling of all rats were measured by two groups of relatively independent experimenters. The secondary paw swelling of the right-hand paw was calculated as (∆mL = paw volume after modeling − paw volume before modeling). The scoring standard of the arthritis index was determined based on our past research (Li et al., 2018). On the 29th day, 2 h after the end of intragastric administration, all rats were anesthetized with 20% urethane, blood was taken from the abdominal aorta, synovial tissues were taken, and sacrificed by cervical dislocation.

### Culture of MH7A cells

RASFs (MH7A cells) were purchased from Bena Chuang Lian Biotechnology Company. The cells were cultured in DMEM medium containing 5% FBS (Biological Industries, ISR) and 1% penicillin-streptomycin (Beyotime Biotechnology) solution. MH7A cells with good growth status were divided into the normoxia/hypoxia group and normoxia/hypoxia administration group (GE, 50 μM), and the oxygen concentration was 21 and 2% in the normoxia and hypoxia groups, respectively. After each group of cells had grown to the bottom of the dish, the medium was aspirated and rinsed 3 times with cold PBS (9 ml). MH7A cells were immediately quenched with liquid nitrogen to inhibit enzymatic activity. Subsequently, cells were scraped off in 1 ml of cold methanol/H_2_O (2:1, v/v). The cells were then crushed using an ultrasonic crusher (instrument parameters: operation 5 s, operation times: 4, interval 4 s). Subsequently, 1.5 ml of methanol was added and vortexed in an ice water bath for 30 s (repeated 6 times). MTBE/H_2_O (4:1, v/v) was used as a solvent to extract the SPL metabolites from MH7A cells. Each EP tube was vortexed for 30 s in an ice bath and allowed to stand at room temperature for 10 min. After sample centrifugation (4°C, 15,000 rpm, 10 min), the supernatant was transferred to a 1.5 ml EP tube and allowed to stand at −80°C for 24 h. After layering, the supernatant was dried using a vacuum centrifuge dryer and re-dissolved in 200 µL methanol, then centrifuged (4°C, 15,000 rpm, 10 min) and the supernatant was transferred into sample vial.

### Sample preparation

The rats were anesthetized after the last administration, blood was taken from the abdominal aorta and the synovial tissue of the left hind limb was taken out. The blood sample was allowed to stand for 1 h, then centrifuged (4°C, 3,000 rpm, 10 min) and the supernatant was aspirated for later use. After the synovial tissue was taken out, it was quenched with liquid nitrogen immediately, and then thoroughly ground with methanol and transferred into an EP tube. The samples were stored at −80°C. Before analysis, the sample was dried with a vacuum centrifugal dryer, the residue was re-dissolved in methanol, vortexed, and then centrifuged (4°C, 15,000 rpm, 10 min). 100 µL of all samples were taken and placed into different numbered injection vials for subsequent UPLC-Q-TOF/MS analysis. In order to monitor the stability of the instrument during the experiment, 10 µL of each group of samples of serum and synovium were mixed and transferred to a sample vial as a quality control (QC) sample. The QC sample was injected five times before the formal injection and once every four samples in the analysis sequence. The coefficient of variation of each characteristic metabolite in the QC sample should be calculated to be less than 30%.

### UPLC-Q-TOF/MS analysis

All samples were analyzed by using the Acquity UPLC system (WATERS, Milford, United States ) connected to Xevo G2-XS Q/TOF mass spectrometer (ACQUITY, WATERS, United States ). A Phenomenex column (C18, 100 × 2.1 mm, 1.6 μm) was used for sample separation. The mobile phase consisted of solvent A (50% acetonitrile and water containing 0.1% formic acid) and solvent B (95% isopropanol and 5% acetonitrile). The flow rate was 0.1 ml/min. The injection volume of each sample was 1 µL and the column temperature was set to 35°C. Sequence analysis was conducted for each sample with interactive insertion. The mobile phase gradient was set as follows: 10% B at 0–3 min, 10%–30% B at 3–5 min, 30%–60% B at 5–6 min, 60%–80% B at 6–12 min, 80%–90% B at 12–18 min, 90%–100% B at 18–20 min, 100%–20% B at 20–24 min, 10% B at 24–30 min. The mass detection was performed in the positive (ESI^+^) and negative (ESI^−^) ion modes and the parameters were set as follows: source temperature, 120°C (ESI^+^)/110°C (ESI^−^); dry gas temperature, 350°C; capillary voltage, 2.5 kV (ESI^+^)/2.0 kV (ESI^−^); gas flow, 600 L/h; Impact energy voltage, 6 V (low)/20–30 V (high); quality scan range 50–1200 m*/z*. The correction method was used in all analyses to ensure the accuracy and repeatability of quality. Leucine enkephalin (200 ng/ml) was used to establish mass spectrometer calibration and reference mass.

### Metabolomics data processing and analysis

The raw data collected by UPLC-Q-TOF/MS (ESI^+^ and ESI^−^ modes) into the Progenesis QI V2.0 software (Waters Corp., Milford, United States ) using baseline filtering, peak identification and correction functions to obtain a data matrix of retention time (t_R_), mass-to-charge ratio and peak intensity. Multivariate statistical analysis was performed by EZinfo 3.0 software. First, unsupervised principal component analysis (PCA) was used to observe the overall distribution between groups of samples and the stability of the entire analysis process, and then supervised orthogonal partial least squares-discriminant analysis (OPLS-DA) was used to distinguish the overall differences in metabolic profiles between groups and to look for metabolites that differed between groups. The statistically significant differential metabolites (*p* < 0.05, fold changes ≥1.5, CV < 30%, VIP-value>1) and their ion fragments at high and low collision energy were matched with Human Metabolomics Database (HMDB) (https://hmdb.ca/) and self-built database to confirm the differential sphingolipid metabolites. Potential biomarkers associated with GE processing were analyzed by MetaboAnalyst 4.0 (http://www.metaboanalyst.ca) and Kyoto Encyclopedia of Genes and Genomes (KEGG) database (http://www.kegg.jp) and a metabolic network was constructed according to the relationships among the identified potential biomarkers.

### Western blotting

To further explore the mechanisms underlying the effects of GE on sphingolipid metabolism, rat synovial tissues were used to perform Western blotting to validate target proteins of interest. Synovial tissues were dissolved with RAPI buffer (containing 1% PMSF) and incubated in ice bath for 30 min to extract total proteins. Total protein concentration was next determined by BCA protein assay kit (Beyotime Biotechnology Corporation Co., Ltd., Shanghai, China). Equal amounts of protein samples were separated by 10% sodium dodecyl-sulfate-polyacrylamide gel electrophoresis (SDS-PAGE) and transferred to Bio Trace nitrocellulose (NC) by electrophoresis membranes. Membranes were blocked with 5% nonfat milk for 2 h at 37°C and subsequently incubated with specific antibodies recognizing acid-sphingomyelinase (A-SMase) overnight at 4°C, followed by horseradish peroxidase-conjugated secondary antibodies. Bands of interest were detected by enhanced chemiluminescence and analyzed with ImageJ software. The values of each target protein were normalized to *ß*-actin.

### Date analysis

All values in the text and figure are represented by mean ± standard deviation (SD). SPSS 23.0 was used to analyze experimental data and compare the differences between the two groups, using student’s *t*-test and One-way ANOVA test. *p* < 0.05 was considered statistically significant.

## Results

### The therapeutic effect of GE on adjuvant arthritis rats

**FIGURE 2:**
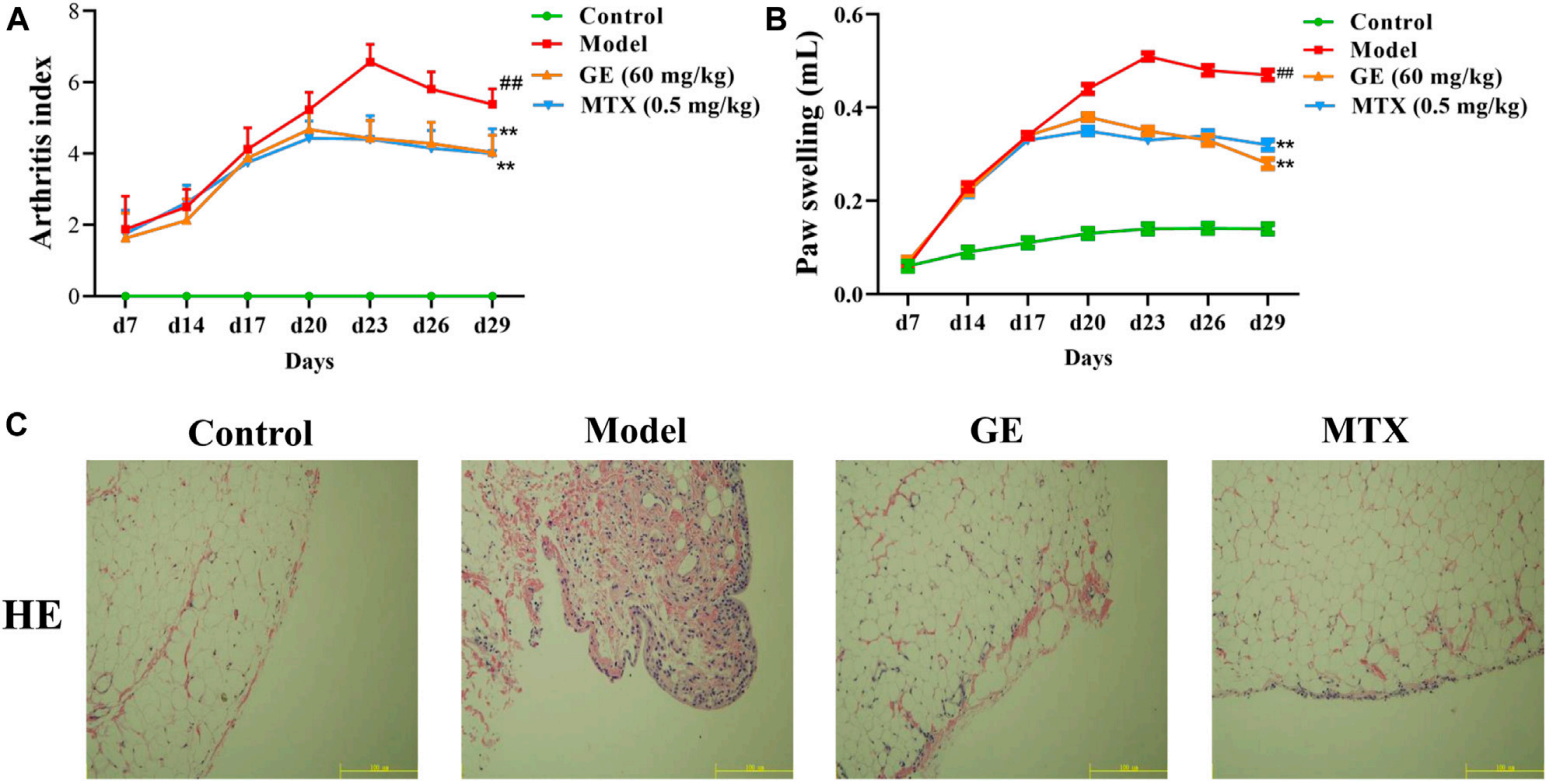
Establishment and evaluation of AA model rats and the therapeutic effect of GE. AA model rats were treated with GE for 14 days. **(A)** The arthritis index of rats in each group was measured, including control group, model group, GE administration group and MTX positive medicine group. **(B)** The degree of secondary paw swelling was measured. **(C)** Histopathological examination of synovial in AA rats. The data represent the means ± SD deviation of six samples in an independent experiment. ^##^ *p* < 0.01, compared with control group; ^**^ *p* < 0.01, compared with model group; *n* = 6.

Studies have shown that the arthritis index and secondary foot swelling of AA rats were significantly higher than those of the normal group, and reached their peak on the 23rd day (Figure 2A,B). The histopathological results of synovial tissue showed that the arrangement of synovial cells in AA rats was disordered, accompanied by inflammatory cell infiltration and the formation of pannus (Figure 2C). These results indicated that we successfully established the AA rat model. Compared with the model group, GE and MTX treatment can inhibited the increase of arthritis index in AA rats, ameliorated secondary foot swelling, reduced the degree of inflammatory cell infiltration, and modified the histopathological changes of the synovium of AA rats. On the 29th day, the GE group had a good therapeutic effect on the reduction of arthritis index and the treatment of secondary foot swelling compared with the MTX group. In summary, these results indicated that GE has a good effect on AA rats.

### Validation of metabolomics methods

**FIGURE 3:**
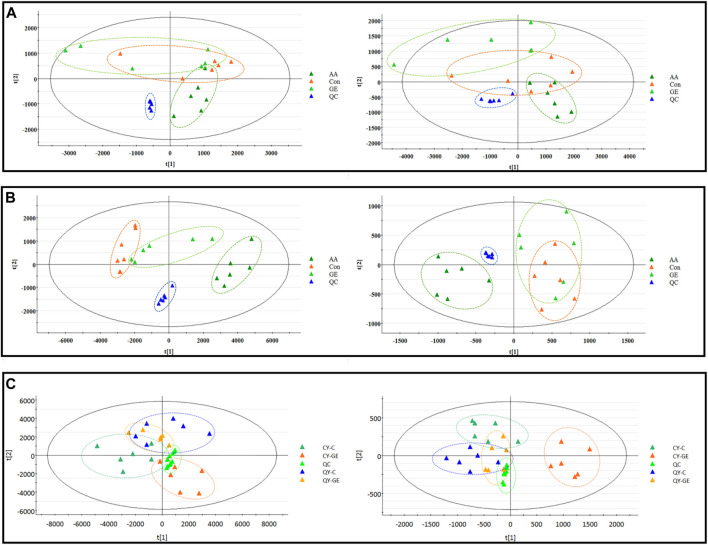
**|** PCA score plots of synovial, serum and MH7A cells extracts in ESI^+^ (left) and ESI^−^ (right) modes. **(A)** PCA score plots of synovial sample; **(B)** PCA score plots of serums sample; AA = model group; C = control group; GE = Geniposide treatment group; QC = quality group; **(C)** PCA score plots of MH7A cells extracts sample; CY-C = normoxia group; CY-GE = normoxia GE treatment group; QY-C = hypoxia group; QY-GE = hypoxia GE treatment group.

The results of the metabolomics experiment depend on whether high-quality experimental data can be obtained. In this study, the stability of the analytical system and samples was tested by periodic injection of QC samples. As shown in Figure 3, the score chart of PCA showed that the clustering of QC samples was obvious in ESI^+^ and ESI^−^ modes. At the same time, we selected a characteristic peak from the chromatogram of each QC sample to obtain the t_R_ and peak area. The relative standard deviation (RSD) of the t_R_ and peak area of these characteristic peaks in the QC samples were 0.50–1.00% and 1.71–5.21% respectively. Therefore, it showed that the established metabonomic analysis method has good stability and reproducibility.

### Effects of synovial inflammation on sphingolipid metabolism in serum, synovium and MH7A cells

**FIGURE 4:**
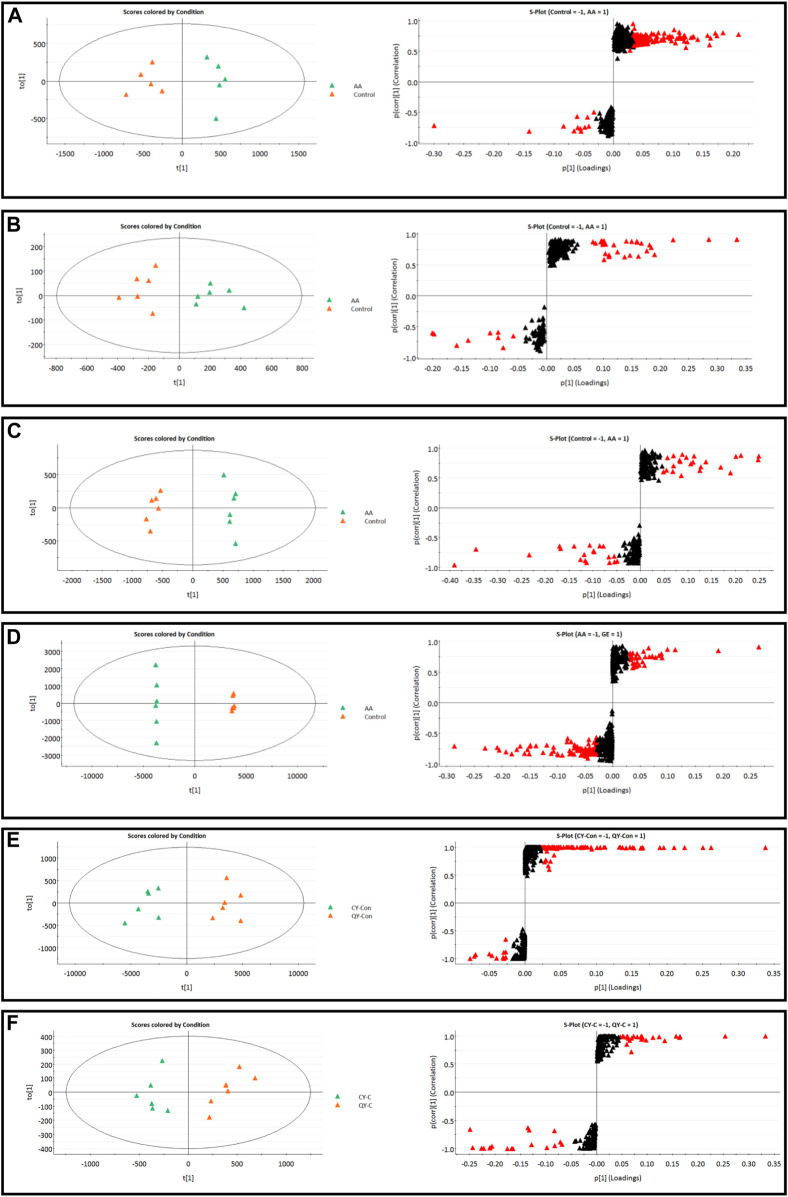
**|** OPLS-DA score plots and S-plots of synovial, serum and MH7A cells samples. **(A)** OPLS-DA model in ESI^+^ mode corresponding to synovial sample (AA vs. C: R^2^Y = 0.93, Q^2^ = 0.85); **(B)** OPLS-DA model in ESI^−^ mode corresponding to synovial sample (AA vs. C: R^2^Y = 0.82, Q^2^ = 0.78); **(C)** OPLS-DA model in ESI^+^ mode corresponding to serum samples (AA vs. C: R^2^Y = 0.99, Q^2^ = 0.96); **(D)** OPLS-DA model in ESI^−^ mode corresponding to serum samples (AA vs. C: R^2^Y = 0.98, Q^2^ = 0.94); **(E)** Corresponding to OPLS-DA model in ESI^+^ mode of MH7A sample (CY-C vs. QY-C: R^2^Y = 0.85, Q^2^ = 0.91); **(F)** Corresponding to OPLS-DA model in ESI^−^ mode of MH7A sample (CY-C vs. QY-C: R^2^Y = 0.84, Q^2^ = 0.88); the variable with VIP >1.0 was highlighted with a red frame in the S-plots of each comparison.

**FIGURE 5:**
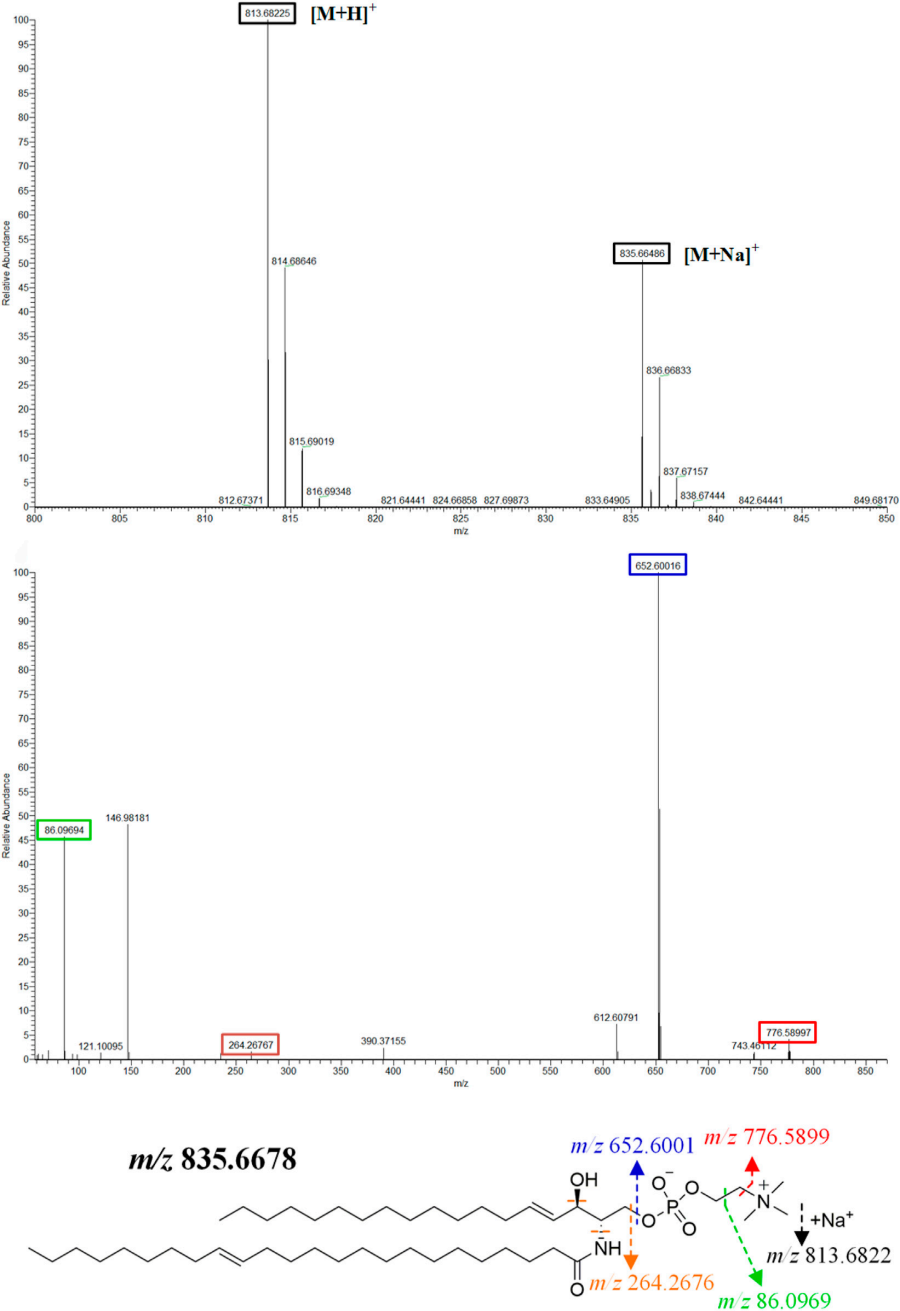
**|** SM (d18:1/24:1 (15Z)) primary and secondary mass spectrometry fragment attribution and cleavage pathways based on UPLC-Q-TOF/MS.

The MS data of serum, synovium and MH7A cells sphingolipid extract were imported into SIMCA-*p* 14.1 software and used PCA to establish a multivariate statistical model for each group of samples. As shown in Figure 3, it was found that there are obvious clusters between the control group and model group in serum and synovium samples, and between the control group and administration group in MH7A samples, and the variability of samples between groups was large. To further reveal the metabolic changes of biological samples in each group and find the related metabolites of RA, the samples were analyzed by OPLS-DA with supervised technology. Generally speaking, the larger the value of R^2^Y and Q^2^ and the closer the ratio is to one, indicates that the established OPLS-DA model is stable and reliable and has a certain prediction ability. The range of R^2^Y value was 0.82–0.99, Q^2^ value was 0.78–0.96, and R^2^Y/Q^2^ value was 0.93–1.09 (Figure 4). In the OPLS-DA model, VIP value >1, *p* < 0.05, fold changes ≥1.5 and CV < 30% were met. By combining Human Metabolome Database, self-built SPLs database and secondary MS fragment analysis, 2, 6 and 7 differential SPLs potential biomarkers were found in serum, synovium and MH7A cells respectively. The provision of precise *m*/*z* data and MS/MS fragmentation data by time-of-flight mass spectrometry, which are helpful identifying differential metabolites induced by AA. Online metabolite databases and self-built databases were applied to identify interesting screening metabolites by accurate *m/z* data and MS/MS spectra. Taking an interesting variable (t_R_-*m/z* 15.78_835.6648) as an example, the quasi-molecular ion *m/z* 835.6648 (ESI^+^) was extracted at 15.78 min in AA rat metabolite profiling. The molecular formula of the metabolite was presumed to be C_47_H_93_N_2_O_6_P by elemental composition analysis using Masslynx 4.1. A search of the 835.6648 m*/z* generated 4 candidate compounds with a mass tolerance of 5 ppm based on the Human Metabolome Database. The characteristic fragmentation ions of *m/z* 835.6648 ([M + Na]^+^) were identified as 813.6822 m*/z* ([M + H]^+^), 776.5899 m*/z* ([M + Na-C_3_H_9_N]^+^), 652.6001 m*/z* ([M + Na-C_5_H_14_NO_4_P]^+^), 390.3715 m*/z* ([M + Na-C_21_H_45_NNaO_5_P]^+^), 264.2676 m*/z* ([M + Na-C_29_H_60_NNaO_6_P]^+^) and 86.0969 m*/z* ([C_2_H_3_N(CH_3_)]^+^) (Figure 5). Among these fragments, those at *m/z* 776.5899, 652.6001, 390.3715, 264.2676 and 86.0969 of SM(d18:1/24:1 (15Z)) were similar to the fragments of SM(d18:1/24:1 (15Z)) in the Human Metabolome Database. Combining the above information, including quasi-molecular ions as well as major fragment ions and the Human Metabolome Database, this AA-induced differential metabolite was annotated as SM (d18:1/24:1 (15Z)). In total, 11 potential sphingolipid biomarkers responsible for the therapeutic effect of GE on RA were screened and identified. Specific information on these potential biomarkers was summarized in Table 1.

**TABLE 1:** **|** Differential sphingolipid metabolites identified in synovial, serum and MH7A cells and GE intervention.

Source	Metabolite	Formula	Retention time (min)	Measured m/z	VIP		Ion Form	HMDB	Trend	
					M/C	GE/M			M/C	GE/M
Synovial	CerP (d18:1/22:0)	C_40_H_80_NO_6_P	15.55	702.5745	1.12	2.33	[M + H] ^+^	HMDB0010703	↑	↓
	LysoSM(d18:0)	C_23_H_51_N_2_O_5_P	16.60	933.7177	1.62	1.50	[2 M + H] ^+^	HMDB0012082	↑	↓
Serum	Araliacerebroside	C_40_H_77_NO_10_	13.78	732.5556	1.15	1.06	[M + H] ^+^	HMDB0033621	↑	↓
	SM(d18:1/18:0)	C_41_H_83_N_2_O_6_P	14.30	731.6073	1.47	1.65	[M + H] ^+^	HMDB0062559	↑	↓
	SM(d18:1/24:1 (15Z))	C_47_H_93_N_2_O_6_P	15.78	813.6822	3.74	2.15	[M + H] ^+^	HMDB0012107	↓	↑
	SM(d18:1/22:0)	C_45_H_91_N_2_O_6_P	15.87	787.6710	2.37	2.31	[M + H] ^+^	HMDB0012103	↓	↑
	SM(d18:1/23:0)	C_46_H_93_N_2_O_6_P	16.28	801.6855	1.97	1.85	[M + H] ^+^	HMDB0012105	↓	↑
	LysoSM(d18:0)	C_23_H_51_N_2_O_5_P	16.60	933.7177	1.34	1.62	[2 M + H] ^+^	HMDB0012082	↑	↓
MH7A	Cer(d18:0/14:0)	C_32_H_65_NO_3_	11.04	512.5046	1.13	2.39	[M + H] ^+^	HMDB0011759	↑	↓
	Cer(d18:0/16:0)	C_34_H_69_NO_3_	11.73	540.5362	1.26	2.67	[M + H] ^+^	HMDB0011760	↑	↓
	Palmitoyl sphingomyelin	C_39_H_79_N_2_O_6_P	13.58	703.5761	1.41	4.16	[M + H] ^+^	HMDB0061712	↑	↓
	Araliacerebroside	C_40_H_77_NO_10_	13.78	732.5556	5.56	3.56	[M + H] ^+^	HMDB0033621	↑	↓
	SM(d18:1/18:0)	C_41_H_83_N_2_O_6_P	14.36	731.6075	1.64	1.64	[M + H] ^+^	HMDB0012089	↑	↓
	Galactosylceramide (d18:1/20:0)	C_44_H_85_NO_8_	15.75	756.6320	1.37	-	[M + H] ^+^	HMDB0010710	↓	-
	LysoSM(d18:0)	C_23_H_51_N_2_O_5_P	16.67	933.7177	1.35	9.75	[2 M + H] ^+^	HMDB0012082	↑	↓

### Intervention mechanism of GE on sphingolipid metabolism in adjuvant arthritis rats and MH7A cells

**FIGURE 6:**
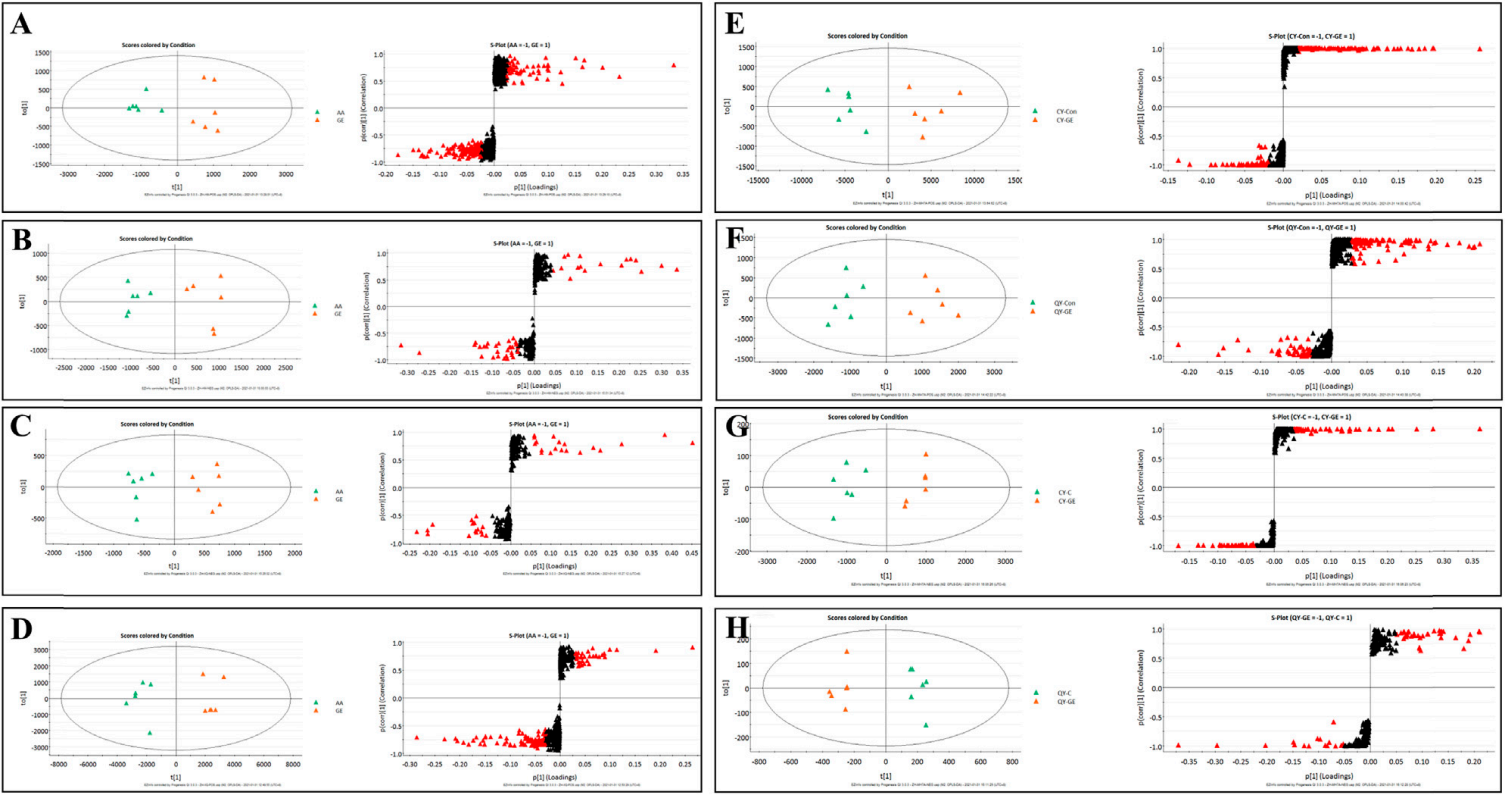
OPLS-DA score plots and S-plots of synovial, serum and MH7A cells samples after GE intervention. **(A)** OPLS-DA model in ESI^+^ mode corresponding to synovial sample (GE vs. AA: R^2^Y = 0.92, Q^2^ = 0.85); **(B)** OPLS-DA model in ESI^−^ mode corresponding to synovial sample (GE vs. AA: R^2^Y = 0.84, Q^2^ = 0.79); **(C)** OPLS-DA model in ESI^+^ mode corresponding to serum samples (GE vs. AA: R^2^Y = 0.95, Q^2^ = 0.81); **(D)** OPLS-DA model in ESI^−^ mode corresponding to serum samples (GE vs. AA: R^2^Y = 0.93, Q^2^ = 0.86); **(E)** and **(F)** Corresponding to OPLS-DA model in ESI^+^ mode of MH7A cells sample (CY-GE vs. CY-C: R^2^Y = 0.85, Q^2^ = 0.77; QY-GE vs. QY-C: R^2^Y = 0.80, Q^2^ = 0.82); **(G)** and **(H)** Corresponding to OPLS-DA model in ESI^−^ mode of MH7A cells sample (CY-GE vs. CY-C: R^2^Y = 0.75, Q^2^ = 0.82; QY-GE vs. QY-C: R^2^Y = 0.88, Q^2^ = 0.91); the variable with VIP >1.0 was highlighted with a red frame in the S-plots of each comparison.

**FIGURE 7:**
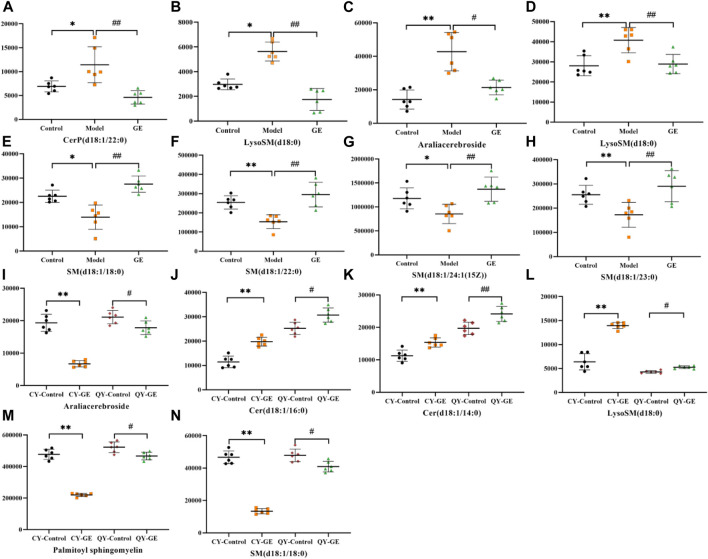
Effects of GE on the standardized abundance of 14 differential metabolites in synovial tissue **(A,B)**, serum **(C ∼ H)** and MH7A cells **(I ∼ N)**. Dates are represented as mean ± SD (n = 6). (In A ∼ H, ^*^ *p* < 0.05, ^**^ *p* < 0.01 compared to the control group; ^#^ *p* < 0.05, ^##^ *p* < 0.01 compared to the model group; In I ∼ N, ^*^ *p* < 0.05, ^**^ *p* < 0.01 compared to the CY-control group; ^#^ *p* < 0.05, ^##^ *p* < 0.01 compared to the QY-control group).

Following the same data processing strategy, we compared the metabolic spectra of synovium and serum of AA rats and MH7A cells in normoxia group and hypoxia group with those obtained by GE administration group, respectively. As shown in the PCA score diagram (Figure 3), it was found in the synovium and serum sample spectra that the data matrix of GE group was more similar to that of the control group than that of the model group. It showed that GE could correct the metabolic disorder of SPLs involved in AA to a certain extent. In each sample group of MH7A cells, it was found that the hypoxia environment can change the metabolic level of SPLs in MH7A cells, while the data matrix of the hypoxia GE group had a slight regression trend compared with the hypoxia group, indicating that GE could callback the metabolite level of endogenous SPLs disturbed by hypoxia to a certain extent. To explore the intervention effect of GE on sphingolipid metabolism of AA rats and normoxia/hypoxia MH7A cells, we performed OPLS-DA statistical analysis on synovial and serum of AA rats model group, administration group and sphingolipid extract of normoxia/hypoxia MH7A cells. Comparing the data from the administration group and model group, there were significant statistical differences between these sphingolipid metabolites (Figure 6). In general, GE significantly recalled 2 (including LysoSM (d18:0) and CerP (d18:1/22:0)) and 6 (including LysoSM (d18:0), SM (d18:1/18:0), Araliacerebroside, SM (d18:1/23:0), SM (d18:1/22:0) and SM (d18:1/24:1 (15Z))) sphingolipid metabolites in synovium and serum samples of AA rats, respectively. In MH7A cells sphingolipid extract, GE could significantly restore the disturbance of 6 (including LysoSM (d18:0), Palmitoyl sphingomyelin, Araliacerebroside, SM (d18:1/18:0), Cer (d18:0/14:0) and Cer (d18:0/16:0)) SPLs differential metabolites in MH7A cells (Figure 7). Their characteristic information was summarized in Table 1. The most notable finding was that the level of Cer decreased significantly after treatment with GE compared with the model group. Therefore, exploring the potential mechanism of the change of Cer level among different groups may promote the therapeutic intervention of RA and better understand the potential therapeutic mechanism of GE.

**FIGURE 8:**
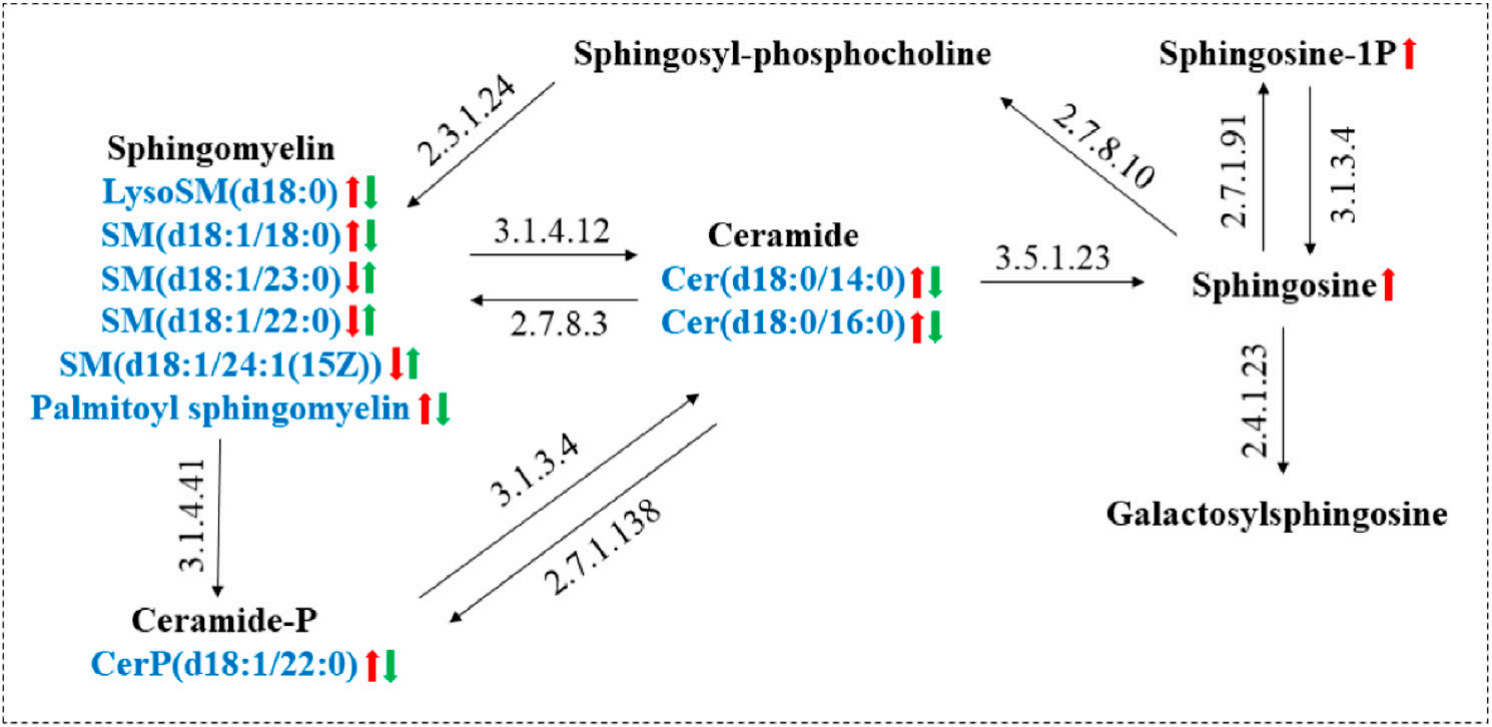
Metabolic networks of major sphingolipid metabolism markers associated with RA and the role of GE intervention. (Red arrows: trends in RA-induced changes in sphingolipid metabolites; Green arrows: trends in sphingolipid metabolites associated with GE treatment).

As shown in Figure 8, The sphingolipid metabolic network was mapped by KEGG to describe the interrelationship between nine biomarkers associated with the interventional effects of GE on AA rats and MH7A cells. Cer, a central component of sphingolipid metabolism, is produced in the inflammatory hypoxia microenvironment mainly by hydrolysis of SM by A-SMase, followed by sphingosine (Sph) production catalyzed by ceramidase (El Kaffas et al., 2018). Sph is further generated by the action of sphingosine kinase 1 (SphK 1) to produce the pro-inflammatory factor SIP (Sun et al., 2020). In our study, Cer levels were restored after GE treatment, which indicated that the mechanism of action of GE intervention in Cer levels required further consideration.

### Effects of GE on sphingolipids metabolizing enzyme Acid-SMase in adjuvant arthritis rats

**FIGURE 9:**
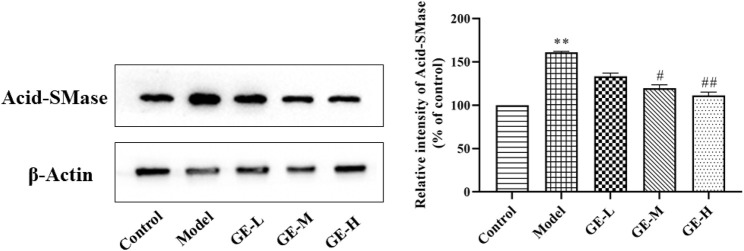
Effect of GE on the expression of Acid-SMase in the synovial tissue of AA rats. The dates are represented as mean ± SD (*n* = 3) ^*^ *p* < 0.05, ^**^ *p* < 0.01 compared to the control group; ^#^ *p* < 0.05, ^##^ *p* < 0.01^#^ compared to the model group, GE-L = GE (30 mg/kg/day, ig), GE-M = GE (60 mg/kg/day, ig), GE-H = GE (120 mg/kg/day, ig).

Ceramides are the central molecules of sphingolipid metabolism (Gomez-Muñoz et al., 2016; Jeffries and Krupenko, 2018). It is now well established that ceramides are produced mainly through *de novo* synthesis initiated by condensation of serine and palmitoyl acyl-CoA or by hydrolysis of membrane sphingolipids catalyzed by acid-sphingomyelinase (A-SMase), and in particular, ceramides produced by A-SMase hydrolysis is involved in more inflammatory processes triggered by pro-inflammatory agonists (Cinque et al., 2003, Kihara, 2016). Previous studies have shown increased expression of A-SMase in the synovium and serum of AA rats, as well as in patients with RA (Miltenberger-Miltenyi et al., 2020). In addition, pharmacological blockade of A-SMase expression reduces ceramides levels and exerts effective anti-inflammatory effects *in vitro* and *in vivo* (Zhao et al., 2021). Thus, we investigated the expression of A-SMase in joint synovial tissues of AA rats in response to GE intervention. As shown in Figure 9, The expression of A-SMase in the model group was significantly higher than that in the control group. Compared with the model group, GE group significantly inhibited the expression of A-SMase in a dose-dependent manner. To sum up, the above results showed the difference in ceramides levels between AA rats and normal rats and the corrective activity of GE, which was associated with the regulation of A-SMase expression.

## Discussion

The FCA-induced AA rat model is a classic model for the treatment of RA, which is characterized by persistent inflammatory hyperplasia of synovial tissue, destruction of cartilage, and progressive swelling of contralateral joints. However, the pathogenesis of RA is extremely complex, involving fibroblast-like synovial cells (FLSs), macrophages, osteoclasts and other cells (Lin et al., 2019). Studies have shown that FLSs are the main cell type of synovial intima, and the change of their biological function plays an important role in the occurrence and development of RA. Dysplastic synovial cells can secrete a large number of inflammatory factors, leading to inflammatory cell infiltration, hypoxia and destruction of the balance of synovial microenvironment, resulting in the aggravation of RA (Du et al., 2019). Therefore, RASFs (MH7A cells) and rats’ synovial tissue were used for metabolic analysis. Previous studies have shown that SPLs are closely associated with the occurrence and development of RA. Among them, the expressions of Cer and S1P were up-regulated, while the expression of SM was down-regulated. During inflammation, the SPLs metabolizing enzyme SMase promotes the hydrolysis of SM into Cer, accelerates the accumulation of Cer, and then induces the release of inflammatory factors such as COX-2 and PGE2, thereby triggering various inflammatory diseases (Qu et al., 2018). To explore whether the positive effects of GE on AA rats and its mechanism are related to the callback of SPLs metabolic disorders, we collected blood and synovial tissues in AA rats for sphingolipid metabolism analysis. The results showed that there are two sphingolipid metabolites LysoSM (d18:0) and CerP (d18:1/22:0) in the synovial samples of AA rats, which are up-regulated to varying degrees compared with the normal group. In serum samples, LysoSM (d18:0), Aralia cerebroside and SM (d18:1/18:0) were up-regulated, SM (d18:1/23:0), SM (d18:1/22:0) and SM (d18:1/24:1 (15Z)) were down-regulated. In the synovial tissues and serum samples of the GE (60 mg/kg) administration group, the contents of the seven sphingolipid metabolites with metabolic disorders were significantly recovered to the normal group’s metabolic level. These results provide the basis for the following view: GE, as the active ingredient of *Gardenia jasminoides Ellis*, has a positive effect on the sphingolipid metabolism disorder in AA rats.

Hypoxia is an important feature of RA. Due to the proliferation of synovial lining and the increase of immune cell infiltration, the demand for oxygen in the synovial is increased. The whole process also involves inflammation, angiogenesis, apoptosis, cartilage degradation and energy metabolism reprogramming (Veale et al., 2017). As the main effector cells of RA, RASFs were cultured in hypoxia to simulate the hypoxia environment in the articular cavity of RA patients. SPLs are important substances that mediate inflammation and other cellular processes. The dynamic balance of their metabolite levels directly affects the course of the disease (Hannun and Obeid, 2018). Sphingomyelinase is one of the key enzymes that control Cer levels in cells. The changes in glycolytic metabolism in RA patients with hypoxia in the articular cavity led to the accumulation of lactate and the appearance of an acidic microenvironment. However, the acidic microenvironment increases A-SMase activity and accelerates SM hydrolysis into Cer, which further promotes the occurrence and development of inflammatory response (Floudas et al., 2020; Insausti-Urkia et al., 2020). To explore the regulatory effect of GE on sphingolipid metabolism disorder of MH7A cells. We extracted SPLs metabolites of MH7A cells under hypoxia (2% oxygen) and normoxia (21% oxygen). The analysis results showed that the levels of Arabia cerebroside, palmitoyl sphingomyelin, lysosm (d18:0), SM (d18:1/18:0), Cer (d18:0/14:0) and Cer(d18:0/16:0) were up-regulated in the hypoxia control group. The level of galactosylceramide (d18:1/20:0) showed a downward trend. After GE (50 µM) intervened in MH7A cells, the levels of the above six up-regulated SPLs metabolites all returned to normal levels and had no significant effect on the level of Galactosyl ceramide (d18:1/20:0). Interestingly, the levels of all three metabolites (Aralia cerebroside, lysoSM (d18:0) and SM (d18:1/18:0)) were up-regulated *in vivo* and *in vitro*. After the intervention of GE administration, they all have recovered to normal levels, so we have reason to infer that GE has a certain regulatory effect on the abnormal metabolism of SPLs in RA.

In living organisms, the hydrophobic skeleton of all complex sphingolipids (SM, cerebroside, ganglioside) is composed of Cer (Castro et al., 2014). Cer is a bioactive metabolite of SPLs family, which is composed of sphingosine bases, but sphingosine bases are mainly composed of 18 carboaminoalcohols covalently bound to a fatty acyl side chain with variable chain length. The number of sphingosine base carbon atoms, the length of fatty acyl side chains and the double bonds at different positions jointly determine the diversity of ceramide structure and function (Clarke, 2018). In eukaryotic cells, Cer is mainly synthesized through two pathways. The first pathway is the *de novo* synthesis pathway that occurs in the endoplasmic reticulum. First, the condensation of serine and palmitoyl Co-A proceeds via catalysis by serine palmitoyl transferase (SPT) and then by ceramide synthase (Cers), which catalyzes the production of Cer. (Gomez-Muñoz et al., 2016). Interestingly, the levels of some enzymes involved in *de novo* ceramide synthesis are up-regulated in inflammatory diseases (Bikman and Summers, 2011). The second pathway is the catabolic pathway of sphingomyelinase, which generates phosphorylcholine and ceramide by hydrolyzing SM present in the plasma membrane or lysosome (Xiang et al., 2021). The most abundant sphingomyelin in mammals is SM, which has great potential to form ceramide. In fact, ceramide produced by sphingomyelinase catalyzed catabolism is involved in a variety of inflammatory processes induced by proinflammatory factors. In particular, proinflammatory factors including HIF-1α, TNF-α, IL-1β, Interferion- γ (IFN- γ) and other cytokines are important stimulators of SMase activity and have been confirmed to be involved in the inflammatory response in different cells (Jenkins et al., 2010). SMase activation is the main metabolic pathway that regulates Cer production during inflammation. The changes in SMase activity caused by hypoxia in different cells and tissues are consistent with the changes in ceramide levels (Cogolludo et al., 2009). In this study, we found that compared with the normoxia group, the levels of sphingolipid metabolites Cer (d18:0/14:0) and Cer (d18:0/16:0) were significantly increased in the hypoxia control group. And after the intervention of GE administration, it showed a downward trend, revealing that GE may achieve the purpose of treating RA by inhibiting the accumulation of Cer in MH7A cells.

## Conclusion

In summary, this study established a lipidomics analysis method based on UPLC-Q-TOF/MS, preliminarily explored the intervention effect of GE on SPLs metabolism in AA rats and the effect of GE on sphingolipid metabolism of MH7A cells in hypoxia microenvironment, and looked for potential biomarkers related to GE treatment. The results showed that except galactosylceramide (d18:1/20:0), GE could recover the change levels of the above 10 sphingolipid biomarkers in varying degrees. According to these different metabolites, GE has a certain regulatory effect on the disorder of sphingolipid metabolism of AA rats and MH7A cells cultured in hypoxia. Western blotting results confirmed that GE-regulated Cer levels were associated with its inhibition of A-SMase expression in synovial tissues of AA rats, which is crucial to reveal the potential anti-inflammatory mechanism of GE in the treatment of RA.

This study used combined metabolomics and lipidomics to explore the mechanism of GE in the treatment of RA for the first time, which may be to restore the disorder of sphingolipid metabolism, provided an experimental basis for the screening of biomarkers for clinical diagnosis of RA, and also offered a strategy for the treatment of RA with TCM.

## Data Availability

The raw data supporting the conclusions of this article will be made available by the authors, without undue reservation.

## Abbreviations

AA, adjuvant arthritis; Cer, ceramide; DSA, d-sphinganine; GE, geniposide; HIF1α, hypoxia-inducible factor-1α; IL-1β, interleukin-1β; IFN-γ, interferon-γ; MTX, methotrexate; OPLS-DA, orthogonal partial least-squares discriminant analysis; PCA, principal component analysis; PEA, palmitoyl ethanolamine; QC, quality control; RA, rheumatoid arthritis; RASFs, rheumatoid arthritis synovial fibroblasts; RSD, relative standard deviation; SM, sphingomyelin; SMase, sphingomyelinase; S1P, sphingosine 1-phosphate; SPL, sphingolipid; TCM, traditional Chinese medicines; TNF-α, tumor necrosis factor-α; UPLC-Q-TOF/MS, ultra-performance liquid chromatography-quadrupole time-of-flight mass spectrometry.
